# Reviewing next of kin regrets in surgical decision-making: cross-sectional analysis of systematically searched literature

**DOI:** 10.1186/s41687-023-00539-1

**Published:** 2023-01-25

**Authors:** Julien Maillard, Tal S. Beckmann, Martin R. Tramèr, Nadia Elia

**Affiliations:** Division of Anesthesiology, Department of Anesthesiology, Pharmacology, Intensive Care and Emergency Medicine, Geneva University Hospitals, Rue Gabrielle-Perret-Gentil 4, 1205 Geneva, Switzerland

**Keywords:** Regret, Next of kin, Surgery

## Abstract

**Background:**

Decision-making concerning relatives undergoing surgery is challenging. It remains unclear to what extent implicated next of kin eventually regret their decisions and how this regret is assessed. Our aim was to systematically review the literature on decisional regret of next of kin and to describe the assessment tools used and the surgical populations studied.

**Methods:**

We included interventional or observational, quantitative or qualitative studies reporting the measurement of decisional regret of next of kin concerning relatives undergoing surgery. We searched a variety of databases without restriction on publication year. We assessed the quality of reporting of quantitative studies using the NIH *Quality Assessment Tool for Observational Cohort and Cross-Sectional Studies* and of qualitative studies using the *Critical Appraisal Skills Program Checklist*.

**Results:**

Thirteen cross-sectional, five prospective cohorts and five qualitative studies matched our inclusion criteria. In 18 studies (78%), patients were children, in five (22%), young or middle-aged adults. No study included elderly or frail patients. Thirteen studies (57%) used the original Decision Regret Scale which was validated for patients, but not for next of kin. Only 3 of the 18 (17%) quantitative studies and only one of the 4 (25%) qualitative studies were rated as “good” in the quality assessment.

**Conclusion:**

None of the retrieved studies used validated tools to assess the decisional regret of next of kin and none of them examined this issue in elderly or frail surgical patients.

**Supplementary Information:**

The online version contains supplementary material available at 10.1186/s41687-023-00539-1.

## Background

Surgical decision making is often complex, especially for patients for whom surgery means a challenging risk-benefit ratio or for vulnerable populations such as children or elderly and frail patients. Health care providers try to make decisions in line with a patient's values and beliefs. This decision-making process includes the patient, and sometimes also family members or close friends. The implication of next of kin in the surgical decision-making processes for patients undergoing high-risk surgery is therefore now strongly encouraged [1].

Next of kin is variably defined depending on the country and culture concerned [2]. In the United States, next of kin have a legal definition based on closest living blood relative, while in Europe, or the United Kingdom, anyone can be designated as next of kin by the patients themselves [3]. Beyond their recognized implication in the decisions concerning patients undergoing surgery, next of kin can also be an essential backup for the fully autonomous patient in every critical decision making situation [2], as they can be a source of practical, emotional and psychological support for patients facing difficult care options [1, 4]. The social, emotional, and even family burden that weighs on next of kin in this process is manifold and often difficult to identify, assess and calibrate. While studies have looked at the postoperative regrets of implicated patients themselves [5], little is known about the perception, and potential regret, of next of kin regarding their role in this decision-making process.

### Aims and objectives

We set out to investigate whether next of kins’ decisional regret was studied in the context of surgery, and if so, in which surgical populations, and what assessment tools were used. Our primary objective was to describe the populations in which it had been studied, and our second objective was to identify which assessment tool had been used.

## Methods

### Study design and setting

We performed a cross-sectional analysis of systematically searched literature. This study was performed at Geneva University Hospitals, Geneva, Switzerland, starting on the 1st of February 2020. The last literature search update was performed on the 9th of September 2021. We report data following the STROBE recommendations for cross-sectional studies [6]. The study was registered in the Research Registry (www.researchregistry.com) under the reference reviewregistry989.

### Literature search

We searched MEDLINE, CENTRAL, EMBASE, CINHAL, Web of Science and PsycINFO databases using a high-sensitivity and low-specificity search strategy. We combined keywords (“general surgery [MeSH Terms]” OR “surgery” OR “surgical”) AND (“regret” OR “regrets” OR “decisional regret”) AND (“next-of-kin” OR “next of kin” OR “family” OR “families” OR “relatives” OR “relative” OR “parents”) in MEDLINE/PubMed, and adapted keywords according to the database searched. As the term "regret" is commonly used, and often appears in contexts unrelated to the present study subject, we deliberately limited the literature research to Title/Abstract to increase research specificity. We checked the bibliographies of retrieved articles and reviews for additional references. We did not apply any restriction on year of publication. Detailed research strategy can be found as Additional file 1.

### Included studies

All interventional and observational, as well as quantitative and qualitative published studies, were eligible for inclusion if they reported on the measurement of next of kin regret regarding a surgical procedure. We did not consider studies reporting on abortion, sterilization, or organ donation as we judged that the psychological impact and decision-making process could potentially be influenced by factors other than the surgical procedure itself; for instance, by the family structure, partner behavior and direct interest or difficult bereavement [7–9]. We also did not consider studies published as case reports or abstracts only. Articles were not considered if they reported on non-surgical populations (further labelled: wrong population), if they did not report on next of kin regret (wrong outcome), if their design was neither qualitative, cross-sectional, cohort nor interventional (wrong design), or if they were written in a language different from English, French, Spanish, German or Italian (foreign language). Definitions of next of kin were taken as used in the published articles.

We used the free web and mobile app Rayyan to screen and manage the identification of publications corresponding to our eligibility criteria [10]. Rayyan (https://www.rayyan.ai) is a web-based collaborative tool to perform the initial screening of published articles using a process of keywords tagging. One author (JM) screened all retrieved articles based on titles and abstracts and excluded all references that did not adhere to our inclusion criteria. A second author (TSB) checked the inclusion and non-inclusion criteria of all studies retrieved. In case of disagreement, a third author (NE) proceeded to an agreement with the other authors.

### Variables and data management

The primary outcome was the surgical population for which next of kin regret was studied. The secondary outcome, if available, was the assessment tool used to evaluate regret. From each included study, we collected data using a standardized and cloud-shared Excel sheet. We extracted information on journal name, year of publication, first author's name and study design from each article. We extracted the tools (including their cut-offs) used to assess next of kin decisional regret. When patients were individually linked to next of kin (further called: “linked-patient”), we extracted their mean age, population type (children, adults), and the type of surgery performed. We retrieved the definition of next of kin, the number of next of kin approached and included, their response rate, the timing of assessment of decisional regret, the percentage of next of kin expressing decisional regret, and the factors identified as associated with decisional regret.

When required, we contacted the authors of included studies to obtain supplemental relevant information. We asked how they adapted their questionnaires for next of kin. We also requested information on missing data. In the event of no response, the authors were contacted by email up to three times.

### Quality scoring

We assessed included publications regarding their quality of reporting. For quantitative studies, we applied the *Quality Assessment Tool for Observational Cohort and Cross-Sectional Studies* from the National Institute of Health (NIH) [11], for qualitative studies, we used the *Critical Appraisal Skills Program* (CASP) Checklist [12]. Quality in study design or execution was classified as "good", "fair", or "poor". The rating “good" described a study with only minimal risk of bias likely to affect the outcome and whose results were considered valid. The rating "fair" described a study susceptible to some biases that were considered not sufficient to invalidate its results. The rating “poor” described the presence of a significant risk of bias. An appropriate duration of follow-up was defined by us as > 30 days. In case of disagreement between the first (JM) and second (TSB) author, we sought an agreement with the input of the last author (NE).

### Bias

We minimized selection bias by using extended keywords and Boolean terms (operator words used in a search like “AND”, “OR”, and “NOT”) in multiple databases with a limited language restriction. We minimized observer bias by having the first and second authors blindly check retrieved data from all included articles.

### Study size

Before the literature search, we were unable to estimate the number of studies reporting on decisional regret of next of kin. We assumed that a minimal number of five peer-reviewed publications were necessary to proceed with the analyses. This minimum of five publication was a purely arbitrary decision. We did not intend, in this descriptive study, to check any specific hypotheses.

### Quantitative variables

We dichotomized studies according to the age of the linked-patients (pediatric: < 18 years; adults: ≥ 18 years). We categorized next of kin into one of three categories: parents or legal guardians (either mother, father, or both), family members (partner or children) or other next of kin. Surgical interventions performed on linked-patients were grouped together into broader categories according to anatomical sites involved. For example, the insertion of a ventilation tube and adenotonsillectomy or tympanoplasty were all categorized into the “ear-nose-throat” (ENT) category. Moreover, we classified surgical interventions according to the surgical risk category (minor, moderate and major) [13].

### Statistical methods

This is a descriptive study, therefore no statistical tests are applied. We provide a description of the identified studies, detailing the different populations of next of kin, linked-patient, surgeries, tools used to assess the decisional regret, cut-off used, and method and timing of assessments. We use numbers and percentages to describe categorical variables and mean and standard deviations or median and inter-quartile ranges for continues variables, as reported in the original reports. Additionally, we provide a detailed narrative description of the tools used to assess decisional regret of next of kin, as well as the cut-offs used to define regret intensities, when reported. Finally, we report on the number and proportion of next of kin experiencing decisional regret across the studies and factors found to be associated with regret in original studies.

### Important changes to methods after study commencement

After protocol registration, we re-examined our initial concerns about the inclusion of studies on abortion, sterilization and organ donation, and eventually decided to consider studies on sterilization (N = 3) but not those on abortion or organ donation.

## Results

### Participants

**Fig. 1:**
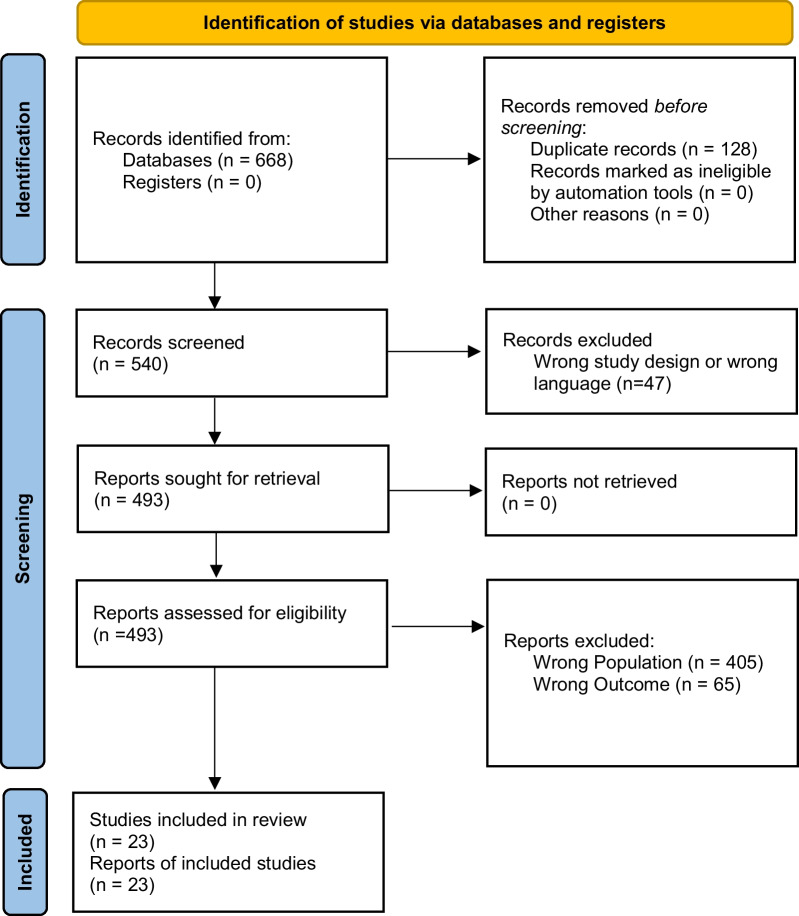
Flow diagram of identified and analysed studies

We screened 540 potentially relevant studies (from inception to year 2021). After excluding studies not matching inclusion criteria we eventually included 23 studies (period from 2009 to 2021) reporting on 5522 next of kin. The full details are described in the flow chart (Fig. 1).

### Descriptive data

Of these 23 articles, 13 were cross-sectional studies, five were prospective cohorts, and five were qualitative studies (Table 1). All studies were published between 2009 and 2021, and originated from ten countries: United States of America (11 studies), Canada (3), Australia (2), United Kingdom (1), Italy (1), Turkey (1), Pakistan (1), Netherlands (1), Belgium (1) and Switzerland (1). Sample sizes ranged from 14 to 1235 approached next of kin (median 106, IQR 49 – 230), and the duration of follow-up for the assessment of decisional regret ranged from 12 days to 9 years (Table 2). All but one [14] study reported only on elective surgeries. Three studies described oncologic patients [15–17].

**Table 1:** Included studies, demography and design

First author	Publication year	Study design	Surgery (procedure*)*	Surgery (minor, moderate or major)	Next of kin			Quality assessment		References
					Number approached	Number analyzed	Relationship to linked-patient	Tool	Rating	
*Adult studies*										
Blumenthal-Barby	2015	Qualitative	Cardio-thoracic *(left ventricular assist device (LVAD) placement)*	Major	15	13	Family member	CASP	Poor	[24]
Gullick	2009	Qualitative	Cardio-thoracic *(Lung volume reduction)*	Moderate	14	14	Family member	CASP	Poor	[33]
Lillie	2014	Cross-sectional	Oncology *(Mastectomy double mastectomy or lumpectomy)*	Moderate	708	517	Family member	NIH	Fair	[15]
Sahgal	2020	Cross-sectional	Neurosurgery *(Brain surgery)*	Major	73	48	Not specified	NIH	Fair	[14]
Schulman-Green	2020	Qualitative	Oncology *(Mastectomy Lumpectomy)*	Moderate	20	20	Family member or friend who helped with treatment decision	CASP	Good	[16]
*Pediatric studies*										
Bethell	2020	Cross-sectional	Urology *(Hypospadia)*	Minor	908	340	Parents	NIH	Good	[18]
Carr	2016	Prospective cohort	ENT *(Tonsillectomy* ± *adenoidectomy)*	Minor	102	94	Parents	NIH	Fair	[19]
Carr	2017	Cross-sectional	ENT *(Ventilation tube insertion)*	Minor	210	210	Parents or legal guardians	NIH	Fair	[23]
Chan	2019	Qualitative	Urology *(Hypospadia)*	Minor	17	9	Parents	CASP	Fair	[31]
Crombag	2021	Qualitative	Fetal surgery (*Spina bifida*)	Moderate	47	29	Parents	CASP	Fair	[35]
Ellens	2017	Prospective cohort	Urology *(Genitoplastia)*	Minor	51	45	Parents	NIH	Fair	[27]
Ghidini	2016	Cross-sectional	Urology *(Hypospadia)*	Minor	744	323	Parents	NIH	Fair	[26]
Hong	2017	Prospective cohort	ENT *(Adenotonsillectomy, tonsillectomy or tympanoplasty with tube insertion)*	Minor	126	64	Parents	NIH	Good	[21]
Hong	2016	Prospective cohort	ENT *(Ear otoplasty)*	Minor	65	62	Parents	NIH	Good	[20]
Javaid	2020	Cross-sectional	Urology (*Hypospadia*)	Minor	91	50	Parents	NIH	Fair	[30]
Lonner	2020	Cross-sectional	Orthopedic *(Posterior spinal fusion)*	Moderate	44	44	Parents	NIH	Fair	[36]
Lorenzo	2014	Prospective cohort	Urology *(Hypospadia)*	Minor	200	116	Parents	NIH	Fair	[22]
Meenakshi-Sundaram	2018	Cross-sectional	Digestive *(Malone Antegrade Continence Enema)*	Moderate	114	**	Family member	NIH	Fair	[28]
Neuhaus	2020	Cross-sectional	Dermatology *(Congenital melanocytic nevi removal)*	Minor	249	219	Parents	NIH	Fair	[17]
O'Loughlin	2013	Cross-sectional	Digestive *(Fundoplication)*	Moderate	122	89	Parents or Family member	NIH	Poor	[32]
Özveren	2016	Cross-sectional	Urology *(Circoncision)*	Minor	1235	623	Parents	NIH	Poor	[34]
Szymanski	2018	Cross-sectional	Urology *(Genital restoration surgery)*	Minor	106	39	Parents	NIH	Poor	[25]
Van Engelen	2021	Cross-sectional	Urology (*Hypospadia*)	Minor	261	97	Parents	NIH	Fair	[29]

ENT, ear-nose-throat; NIH, National Institute of Health—Quality Assessment Tool for Observational Cohort and Cross-Sectional Studies; CASP, Critical Appraisal Skills Programme Checklist Studies

^**^Results of patients and next of kin are not separated

**Table 2:** Decisional regret assessment tools and reported results

First author	Publication year	Decisional regret assessment				Reported results		
		Tool used	Categorisation of scale	Method of administration	Timing (months)	Next of kin with DR [n/N, (%)]	Next of kin with strong DR [n, (%)]	DRS score
*Adult studies*								
Blumenthal-Barby	2015	DRS	Not reported	Face-to-Face or Phone	Not reported	1/13 (8)	Not reported	1.48 (0.78)^a^
Gullick	2009	Open question	Not applicable	Face-to-Face	6 and 12	1/14 (7)	Not applicable	Not applicable
Lillie	2014	DRS-adapted	Unclear	Mail	60 (not reported)^a^	114/517 (22)	Not applicable	Not applicable
Sahgal	2020	DRS-adapted	Not reported	Face-to-Face or Mail	0.4	Not reported	Not reported	12.5 (0–31)^b^
Schulman-Green	2020	Structured interview	Not applicable	Face-to-Face	Not reported	Not reported	Not applicable	Not applicable
*Pediatric studies*								
Bethell	2020	DRS	1–25 mild DR ≥ 26 moderate to strong DR	Online Survey	15.5 (9.6–21.1)^b^	154/340 (45)	21 (6)	Not reported for the entire sample
Carr	2016	DRS	0 no DR ≥ 26 moderate DR	Face-to-Face	1 to 3	55/94 (59)	9 (10)	8.78 (not reported)^a^
Carr	2017	DRS	≥ 26 moderate DR	Face-to-Face	13 (0.05 to 111)^a^	62/210 (30)	16 (8)	6.98 (13.74)^a^
Chan	2019	Closed question	Not applicable	Face-to-Face	< 6	0/9 (0)	Not applicable	Not applicable
Crombag	2021	Structured interview	Not applicable	Face-to-Face	3 to 6	0/29 (0)	Not applicable	Not applicable
Ellens	2017	DRS	1–25 mild DR ≥ 26 moderate to strong DR	Not reported	12	12/45 (27)	3 (7)	Not applicable
Ghidini	2016	DRS	1–25 mild DR ≥ 26 moderate to strong DR	E-mail	60 (15–110)^c^	297/323 (92)	128 (40)	Not applicable
Hong	2017	DRS	1–25 mild DR ≥ 26 moderate to strong DR	Face-to-Face	10 to 16	28/64 (44)	1 (2)	0 (0–15)^b^
Hong	2016	DRS	1–25 mild DR ≥ 26 moderate to strong DR	Face-to-Face	6	24/62 (39)	2 (3)	0 (0–5)^b^
Javaid	2020	DRS	1–25 mild DR ≥ 26 moderate to strong DR	Phone	Not reported	27/50 (54)	5 (10)	17.4 (21.8)^a^
Lonner	2020	Addition of a question on regret to the Patient Generated Index	≥ 1 DR (continuous scale from 1 to 10)	Not reported	Preoperative (anticipated regret)	Not applicable	Not applicable	Not applicable
Lorenzo	2014	DRS	1–25 mild DR ≥ 26 moderate to strong DR	Face-to-Face	12	58/116 (50)	10 (9)	8.9 (12.3)^a^
Meenakshi-Sundaram	2018	DRS	1–25 mild DR ≥ 26 moderate to strong DR	Not reported	49	Regret of patients and next of kin are combined	Not reported	7 (12)^a^
Neuhaus	2020	7 items, 4 point-Likert scale	Likert scale from 1 "strongly disagree" to 4 "stongly agree"	Online Survey	Not reported	97/219 (44)	Not reported	Not applicable
O'Loughlin	2013	Closed question	Not applicable	Face-to-Face or Phone	77 (43–89)^b^	2/89 (2)	Not applicable	Not applicable
Özveren	2016	Open question	Not applicable	Online Survey	0 to 60	3/623 (0.5)	Not applicable	Not applicable
Szymanski	2017	DRS	1–25 mild DR ≥ 26 moderate to strong DR ≥ 51 strong DR ≥ 76 very strong DR	Online Survey	53 (not reported)^b^	8/39 (21)	0	5 (not reported)^a^
Van Engelen	2021	DRS	1–25 mild DR ≥ 26 moderate to strong DR	Online Survey	85.2 (28.8)^a^	49/97 (51)	11 (11)	9.7 (12.6)^a^

References are listed in alphabetical order (first author's name)

DR: Decisional Regret; DRS: Decision Regret Scale; DRS-adapted: Decision Regret Scale adapted by authors for next of kin

^a^Mean (sd); ^b^median (IQR), ^c^median (range)

### Quality assessment

Three of the 18 quantitative studies were rated as “good”, 12 as “fair” and three were considered of “poor” quality. One of the five qualitative studies was rated as “good”, two as “fair”, and two were considered “poor” (Table 1).

The main reasons for downgrading quantitative studies from “good” to “fair” were the absence of a sample size justification or lack of power, inappropriate duration of follow-up, or more than 20% loss to follow-up. Qualitative studies were downgraded due to a lack of description of the relationship between researchers and participants or insufficient description of data analyses.

### Outcome data and main results

#### Populations and settings

In 18 studies (78%), patients were children and in the remaining five, they were young or middle-aged adults. None included specifically elderly or frail patients. Patients underwent urological (9 studies), ear-nose-throat (4), digestive (2), oncological (2), cardio-thoracic (2), orthopedic (1), neurosurgical (1) or fetal surgery (1), or dermatological (1) interventions. Two surgeries were categorized as major, seven as moderate and 14 as minor surgeries. In the 18 studies involving children, next of kin were defined as parents or legal guardians (15 studies), or as parents or family members (2). In one, next of kin were undefined. In the five adult studies, next of kin were defined a family member (4 studies) or as family members or friends over the age of 18, or as “people able to communicate in English” (1) (Table 1).

In none of the studies, the number of included next of kin was clearly reported. Most of them only reported on the number of next of kin who were successfully contacted, but not the number of those who were actually contacted. Furthermore, none of the reports included a flow chart. The proportion of next of kin agreeing to participate, among those successfully contacted, ranged between 42% to 100%.

We contacted six authors for further information. Despite three subsequent e-mail contacts, none of them responded.

#### Tools used to assess decisional regret

A large variety of tools to measure regret was used in these studies (Table 2). Thirteen studies [18–30] used the 5 items Decision Regret Scale, ranging from 0 (no regret) to 100 points (highest regret), which has been validated in patients only [5]. Two studies each used a modified but non-validated Decision Regret Scale that had been adapted for next of kin by the authors (Table 3) [14, 15], one closed question to assess decisional regret [31, 32], one open question [33, 34], or a qualitative structured interview [16, 35]. Finally, one study each used a 4-point Likert scale similar to the Decision Regret Scale but with 7 questions instead of 5 [17], or an adapted quality-of-life questionnaire (Patient Generated Index) [36].

**Table 3:** Decisional Regret Scale, original and adapted versions

	Original	Adapted	
	Brehaut et al. [38]	Lillie et al. [15]	Sahgal et al. [14]
Items^a^			
1	It was the right decision	I wish she would have made a different decision about what type of surgery to have	It was the right decision
2	I regret the choice that was made	I wish she would have chosen a different surgeon to perform her surgery	I regret the choice that was made
3	I would go for the same choice if I had to do it over again	I wish she would have taken more time to make decision about her treatment	I would go for the same choice if I had to do it over again
4	The choice did me a lot of harm	I wish she would have consulted more doctors about her treatment before making a decision	*(item intentionally removed)*
5	The decision was a wise one	I would have her do everything the same	The decision was a wise one

^a^Likert-scale (1 to 5): strongly agree—agree—neither agree nor disagree—disagree—strongly disagree

### Methods of assessment

Decisional regret was assessed face-to-face (9 studies), as online survey (5), face-to-face or by phone (3), face-to-face or by mail (1), by mail only (1), or by email (1). Three studies did not report on a method of assessment (Table 2) [27, 28, 36].

In 18 studies, the proportion of next of kin reporting on decisional regret ranged from 0 to 59%. Most regrets were rated as mild (Decision Regret Scale < 26). Ten studies reported cases of moderate to strong regret (Decision Regret Scale > 26), with incidences ranging from 2 to 17% of next of kin studied (Table 2).

One article compared the regret of parents of children whose nevus was, or was not removed [17]. Significantly less regret was expressed by next of kin of children who had their nevus removed compared with those who had not, but no difference in regret was found between the two populations of children. Two other studies compared decisional regret of next of kin of adult patients [24, 28]. In both studies, decisional regret of next of kin was similar to the decisional regret of the linked patients.

Some studies described factors that the authors judged as potentially associated with decisional regret of next of kin, either specific or non-specific to the surgery. For example, preoperative decisional conflict, the presence of postoperative complications, and poor involvement in decision making were identified as factors that could increase decisional regret of next of kin. However, most of the results should be interpreted with caution due to limitations inherent to the study designs used (Additional file 2: Appendix).

## Discussion

### Key results

Three main results emerge from this analysis. Firstly, and despite a comprehensive search strategy, we retrieved only 23 relevant studies investigating decisional regret of next of kin in the perioperative setting. Second, less than one-quarter of the retrieved studies dealt with next of kin of adult patients, and, perhaps most importantly, none of the adult studies included specifically elderly or frail patients. Finally, all these studies used unvalidated self-developed tools or a decision regret scale that has been validated for patients only.

### Interpretation

Decisional regret of patients having undergone surgery has been investigated for more than 30 years [37] and the Decision Regret Scale, a tool to assess decisional regret of patients, has been validated almost 20 years ago [38]. A systematic review, published in 2017 and including more than 70 articles, reported on perioperative regret in 15% of adult patients [5]. The limited number of retrieved studies dealing with decisional regret of next of kin of adult surgical patients was unexpected. Not surprisingly, we found more relevant data for the pediatric population as family members are routinely involved in the pediatric decision making process [39]. The lack of information from the literature on decisional regret of next of kin dealing with adults undergoing surgery is difficult to explain and cannot be satisfactory. Moreover, given the evolution of medical demography over the last decades, it seems crucial to obtain data concerning one particular adult population, the elderly and frail. Indeed, according to the United Nations, the population aged 80 and over is expected to increase more than threefold by 2050 [40]. Due to their comorbid chronic diseases and disabilities, most of these elderly people are categorized as frail and, each year, a growing number of them undergo high-risk surgery [41], with an increased probability of perioperative morbidity and mortality [42–44].

The surgical and perioperative literature demonstrates a growing interest in the implication of next of kin in surgical decision making, especially for the elderly or frail patients undergoing high-risk procedures, since it provides an opportunity to consider, and to anticipate, together with the patient, and his/her physician and family, unwanted outcomes before they arise [45, 46]. It has been suggested that next of kin engagement was crucial for oncological patients, particularly for those undergoing surgical resection of late-stage diseases [1]. A recent study identified a need for more information and decisional support of next of kin during preoperative discussions, including clarification of treatment options, of postoperative expectations and of advanced care planning [45]. Preoperative advance care planning discussed with both patient and family may avoid undesired life-sustaining treatment in case of severe complications [47]. Recently, Steffens et al developed a prompt question list with patients and their families to address the decisional and informational needs of surgical patients [45]. The purpose of the list was to improve patient engagement and to reduce postoperative regret or conflict about postoperative treatments. However, the corresponding validation study found no difference in primary patient outcomes and regret of next of kin was insufficiently reported to draw any conclusions [48]. To date, not only are preoperative shared discussions inconsistently used [47], but it remains unclear how next of kin of relatives undergoing surgery are informed, on what basis they take decisions, whether these match the patient’s will and perhaps influence their decisions, and how they deal with uncertainty and potential regret once they have taken a decision.

### Limitations

There are some limitations related to our analysis. First, we identified only a limited number of articles dealing with decisional regret of next of kin caring for relatives undergoing surgery and the studies identified were of low quality and small sample sizes. We may have missed relevant studies due to our high specificity search strategy. Second, we decided to exclude studies on abortion and organ donation as we assumed that, in these specific situations, the potential regret expressed by next of kin and its influence on the decision-making process may be related to circumstances beyond the surgical procedure itself. We initially retrieved three studies [49–51] on sterilization but decided not to include them as next of kin were the partners of the patients, and were therefore fully integrated as a complementary entity into the decision process, being directly affected by the risk and benefit of the procedure regarding the ability to reproduce. And third, most studies searching for factors associated with decisional regret failed to report on the baseline risk of regret of next of kin. This made the clinical relevance of the reported odds or risk ratios unclear.

Additionally, we reported the severity of regret according to the Decision Regret Scale when the latter was available. This severity classification was first published by Sheehan et al. [52], and then subsequently taken up by many authors. However, to our knowledge, there is surprisingly no publication justifying this classification.

## Research agenda

Our study highlights several issues that may inform future research. For instance, about half of the studies were using the original Decision Regret Scale, which was validated for patients only but not for next of kin [38]. In two studies, the authors modified the original scale to assess regret in next of kin [14, 15]. The other studies were using self-invented scores.

The limited number of studies addressing regret of next of kin in the surgical setting may be related to the lack of a validated tool to assess decisional regret, and is unsatisfactory. We still do not know how frequent and critical decisional regret of next of kin may be in the surgical setting and to what extent this regret is associated with external factors (i.e., age, surgery, timing, etc.). In particular, there is a lack of data on decisions taken regarding elderly or frail patients. This information is needed to inform our understanding of the implication of next of kin in both the preoperative decision-making process and the postoperative management of high-risk patients. Further research is needed to develop, and to validate, tools to assess decisional regret in next of kin. Our study shows the limitations of published measurement tools and supports the need for a systematic concept elicitation process that follows recent FDA guidelines [53]. Such a tool would open a broad research area for surgical and perioperative care of high-risk surgical patients. It would allow the formal identification of factors associated with next of kin decisional regret and potential interventions to reduce it. These data may also lead to improved shared decision-making and a more reliable patient-next of kin-physician relationship [5]. A validated decision regret tool for next of kin is thus the mandatory first step of a critical research area.

## Conclusions

Our study suggests that the potential decisional regret of next of kin regarding their relatives undergoing surgery has not yet been sufficiently examined in good quality studies, especially, concerning elderly or frail patients undergoing high-risk major surgeries. Moreover, it identified a lack of validated tools for the assessment of decisional regret of next of kin in the context of surgery. Such tools are urgently needed and should be able to identify and critically discriminate potential constraints affecting decisional regret.

## Supplementary Information

**Additional file 1:** Detailed research strategy.

**Additional file 2: Appendix.** Factors associated or not associated with decisional regret, and degree of evidence (quantitative studies).

## Data Availability

Detailed research strategy can be found as Additional file 1 and additional data are available from the corresponding author on reasonable request.

## Abbreviations

NIH: National Institute of Health
CASP: Critical Appraisal Skills Program
ENT: Ear-nose-throat
IQR: Interquartile range
