# Supplementary material for: Reviewing next of kin regrets in surgical decision-making: cross-sectional analysis of systematically searched literature
Source: J Patient Rep Outcomes. 2023 Jan 25;7:5. doi: 10.1186/s41687-023-00539-1 (PMC9877257; doi:10.1186/s41687-023-00539-1)
Supplement: Supplementary file 1 — Additional file 1: Detailed research strategy. [file 41687_2023_539_MOESM1_ESM.pdf]

Date	Database	Keywords	Limitations	Retrieved
25.02.2020	Pubmed	(general surgery[MeSH Terms] OR surgery OR surgical) AND (“regret” OR “regrets” OR “decisional regret”) AND (“next-of-kin” OR “next of kin” OR “family” OR “families” OR “relatives” OR “relative” OR “parents”)	Title AND Abstract	303
25.02.2020	CENTRAL	(surgery OR surgical) AND (“regret” OR “regrets” OR “decisional regret”) AND (“next-of-kin” OR “next of kin” OR “family” OR “families” OR “relatives” OR “relative” OR “parents”)	Title AND Abstract AND key words	45
25.02.2020	WebofScience	(surgery OR surgical) AND (“regret” OR “regrets” OR “decisional regret”) AND (“next-of-kin” OR “next of kin” OR “family” OR “families” OR “relatives” OR “relative” OR “parents”)	Topic	83
25.02.2020	PsycINFO	(surgery OR surgical) AND (“regret” OR “regrets” OR “decisional regret”) AND (“next-of-kin” OR “next of kin” OR “family” OR “families” OR “relatives” OR “relative” OR “parents”)	None	44
25.02.2020	CINHAL	(surgery OR surgical) AND (“regret” OR “regrets” OR “decisional regret”) AND (“next-of-kin” OR “next of kin” OR “family” OR “families” OR “relatives” OR “relative” OR “parents”)	Title AND abstract	18
			Total Retrieved	493
			Total after Duplication Removal	399
			Included	15
			Excluded	384
25.02-27.11.2020	Pubmed	(general surgery[MeSH Terms] OR surgery OR surgical) AND (“regret” OR “regrets” OR “decisional regret”) AND (“next-of-kin” OR “next of kin” OR “family” OR “families” OR “relatives” OR “relative” OR “parents”)	Title AND Abstract	18
25.02-27.11.2020	CENTRAL	(surgery OR surgical) AND (“regret” OR “regrets” OR “decisional regret”) AND (“next-of-kin” OR “next of kin” OR ‘ “family” OR “families” OR “relatives” OR “relative” OR “parents”)	Title AND Abstract AND key words	9
25.02-27.11.2020	WebOfScience	(surgery OR surgical) AND (“regret” OR “regrets” OR “decisional regret”) AND (“next-of-kin” OR “next of kin” OR “family” OR “families” OR “relatives” OR “relative” OR “parents”)	Topic	11
25.02-27.11.2020	PsyChinfo	(surgery OR surgical) AND (“regret” OR “regrets” OR “decisional regret”) AND (“next-of-kin” OR “next of kin” OR “family” OR “families” OR “relatives” OR “relative” OR “parents”)	None	26
25.02-27.11.2020	CINHAL	(surgery OR surgical) AND (“regret” OR “regrets” OR “decisional regret”) AND (“next-of-kin” OR “next of kin” OR “family” OR “families” OR “relatives” OR “relative” OR “parents”)	Title AND abstract	2
			Total Retrieved	66
			Total after Duplication Removal	55
			Included	5
			Excluded	50
28.11.2020-08.09.2021	Pubmed	(general surgery[MeSH Terms] OR surgery OR surgical) AND (“regret” OR “regrets” OR “decisional regret”) AND (“next-of-kin” OR “next of kin” OR “family” OR “families” OR “relatives” OR “relative” OR “parents”)	Title AND Abstract	16
28.11.2020-08.09.2021	CENTRAL	(surgery OR surgical) AND (“regret” OR “regrets” OR “decisional regret”) AND (“next-of-kin” OR “next of kin” OR “family” OR “families” OR “relatives” OR “relative” OR “parents”)	Title AND Abstract AND key words	4
28.11.2020-08.09.2021	WebOfScience	(surgery OR surgical) AND (“regret” OR “regrets” OR “decisional regret”) AND (“next-of-kin” OR “next of kin” OR “family” OR “families” OR “relatives” OR “relative” OR “parents”)	Topic	12
28.11.2020-08.09.2021	PsyCInfo	(surgery OR surgical) AND (“regret” OR “regrets” OR “decisional regret”) AND (“next-of-kin” OR “next of kin” OR “family” OR “families” OR “relatives” OR “relative” OR “parents”)	None	12
28.11.2020-08.09.2021	CINHAL	(surgery OR surgical) AND (“regret” OR “regrets” OR “decisional regret”) AND (“next-of-kin” OR “next of kin” OR “family” OR “families” OR “relatives” OR “relative” OR “parents”)	Title AND abstract	65
			Total Retrieved	109
			Total after Duplication Removal	86
			Included	3
			Excluded	83
			TOTAL	
			Total Retrieved	668
			Total after Duplication Removal	540
			Included	23
			Excluded	517

Supplementary Material; Detailled Research Strategy
