# Supplementary material for: Reviewing next of kin regrets in surgical decision-making: cross-sectional analysis of systematically searched literature
Source: J Patient Rep Outcomes. 2023 Jan 25;7:5. doi: 10.1186/s41687-023-00539-1 (PMC9877257; doi:10.1186/s41687-023-00539-1)
Supplement: Supplementary file 2 — Additional file 2: Appendix. Factors associated or not associated with decisional regret, and degree of evidence (quantitative studies). [file 41687_2023_539_MOESM2_ESM.pdf]

First author	Publication Year	N approached/N analyzed	Surgical context	Factors potentially associated with DR in next of kin investigated	Degree of evidence	Reference
**Adult studies**						
Lillie	2014	708/517	Oncology *(mastectomy, double mastectomy or lumpectomy)*	**Being less-acculturated Hispanic versus Caucasian; adjusted for all decision-making process (45%); insufficient information receipt; low involvement;** being African-American; being Hispanic (high acculturated)	Very low	15
Sahgal	2020	73/48	Neurosurgery *(brain surgery)*	Complexity of the decision (transition to comfort measures, DNR order or brain surgery)	Very low	14
**Pediatric studies**						
Bethell	2020	908/340	Urology *(hypospadia)*	**Postoperative complication requiring repeat surgery; Distal hypospadias; Small glans**; Overall cases undertaken per surgeon/per year per surgeon; Age at primary procedure; Meatal position; Shallow glans groove; Small glans; Chordee; Surgical approach; Circumcision at the initial procedure; Two-stage repair; Complications not requiring surgery; Increase in age at primary surgery per month	Very low	18
Carr	2016	102/94	ENT *(tonsillectomy +/- adenoidectomy)*	**Preoperative DC;** Sex; Age; Parental perception of resolution of preoperative complaint; Whether the child had an ER visit; Postoperative hemorrhage; Dehydration; Parental call to the staff in the post-operative period	Very low	19
Carr	2017	210/210	ENT *(ventilation tube insertion)*	**Reason for visit on day of survey; History of reflux;** Asthma; Cleft palet; Trisomia 21; Developmental delay; Gender; Age	Very low	23
Ellens	2017	51/45	Urology *(genitoplastia)*	**Intermediate education degree; Illness uncertainity at baseline; CAH vs distal hypospadias repair;** Surgical complications	Very low	27
Ghidini	2016	744/323	Urology *(hypospadia)*	**Parent educational level; First born; Initial desire to avoid surgery; Familiy history of hypospadias; PPPS score; DVISS score**; Parental age; Parental gender; Desire to preserve the prepuce; Seeking additional medical advice; Child age (surgery and follow-up); Surgical technique; Preputial managment; Length of follow-up; Postoperative complications	Very low	26
Hong	2017	126/64	ENT *(adenotonsillectomy, tonsillectomy or tympanoplasty with tube insertion)*	**Preception of Shared Decision Making quality; Decisionnal conflict scale;** Parent education; Family income; Ethnicity; Child and parent gender/age; Consulting surgeon; Child's previous surgical experience; Previous surgical experience of any child in the family	Very low	21
Hong	2016	65/62	ENT *(ear otoplasty)*	**Postoperative complications; DC as a dichotomous variable (DCS >= 25); Perception of Shared Decision Making quality;** Surgery type; Previous surgical experience; Consulting surgeon; Parent education level; Decision to proceed with surgery; DCS	Very low	20
Javaid	2020	91/50	Urology *(hypospadia)*	**Non educated mothers; Vaginal route of delivery; Development of complications after surgery; Sad or grieved feelings after surgery;** Fathers qualification; Previously circumcised; Any other anomaly in siblings; Family history of hypospadias; Counseling done by resident; Any exposure to drugs during surgery	Very low	30
Lonner	2020	44/44	Orthopedic *(Posterior spinal fusion)*	**Sleep; Uncertainty of future health; Physical appearance**	Very low	36
Lorenzo	2014	200/116	Urology *(hypospadia)*	**Initial DC; Postoperative complications; Parental desire to avoid circoncision;** Parent age; Number of offspring; Number of male siblings; Index case birth order; Familial history of hypospadias; Educational level; Surgery intent before initial appointment and counseling physician training level; Previsit topic research and self-reported excellent knowledge about hypospadias	Very low	22
Meenakshi-Sundaram	2018	114/**	Digestive *(malone antegrade continence enema)*	Not extractable: mixed population of patients and next of kin	Very low	28
Neuhaus	2020	249/219	Dermatology *(congenital melanocytic nevi removal)*	**Older age of the child at the time of the survey;** Socioeconomic status; Male sex of child; TBSA score; Age at first surgery; Total number of surgeries; Complication related to surgery; POSAS overall opinion of the scar	Very low	17
O'Loughlin	2013	122/89	Digestive *(fundoplication)*	None	Very low	32
Özveren	2016	1235/623	Urology *(circumcision)*	**Source of information providers (internet/social media versus health-care providers); Sufficient preprocedural counseling**	Very low	34
Szymanski KM	2018	106/39	Urology *(genital restoration surgery)*	**Children underwent FGRS more recently;** Child's age at FGRS; Preoperative degree of virilization; Being a mother; Undergoing another surgery; Earlier surgery	Very low	25
Van Engelen	2021	261/97	Urology *(hypospadia)*	**DC, Psychosocial behavior problems of the child;** Time since diagnosis; Postoperative complications; Multiple surgeries; Hypospadias phenotype; Satisfaction with the cosmetic result; Parental level of education	Very low	29

Appendix. Factors associated or not associated with decisional regret, and degree of evidence (quantitative studies)

N: numer; DR: decisional regret; DNR: do not ressucitate; DC: decisional conflict; DCS: decisional conflict scale; ENT: ear nose throat; ER: emergency room; CAH: congenital adrenal hyperplasia; PPPS: paediatric penile perception score; DVISS: dysfunctional voiding and incontinence scoring system; TBSA: total body surface area; POSAS: patient and observer scar assessment scale; FGRS: female genital restoration surgery

References are listed in alpabetical order (name of first author). In bold: outcomes reported as associated with DR. In plain: ouctomes reported as not associated with DR.
